# HIV and SIV Envelope Glycoproteins Interact with Glycolipids and Lipids

**DOI:** 10.3390/ijms241411730

**Published:** 2023-07-21

**Authors:** Rémi Planes, Elmostafa Bahraoui

**Affiliations:** INFINITY, INSERM, CNRS, CHU Purpan Toulouse, 31024 Toulouse, France; remiplanes@gmail.com

**Keywords:** HIV receptors, lipids, glycolipids

## Abstract

The present study demonstrates that, in addition to interacting with galactosylceramide (GalCer), HIV-1, HIV-2, and SIV envelope glycoproteins are able to interact with glucosylceramide (GlcCer), lactosylceramide (LacCer), and ceramide. These interactions were characterized by using three complementary approaches based on molecular binding and physicochemical assays. The binding assays showed that iodinated radiolabeled HIV-1 and HIV-2 glycoproteins (^125^I-gp) interact physically with GalCer, GlcCer, LacCer, and ceramide previously separated by thin layer chromatography (TLC) or directly coated on a flexible 96-well plate. These interactions are specific as demonstrated, on the one hand, by the dose-dependent inhibition in the presence of various dilutions of immune, but not non-immune, sera, and, on the other hand, by the absence of interaction of these glycolipids/lipids with ^125^I-IgG used as an unrelated control protein. These interactions were further confirmed in a physicochemical assay, based on the capacity of these glycolipids for insertion in a pre-established monomolecular film, as a model of the cell membrane, with each glycolipid/lipid. The addition of HIV envelope glycoproteins, but not ovomucoid protein used as a negative control, resulted in a rapid increase in surface pressure of the glycolipid/lipid films, thus indirectly confirming their interactions with GalCer, GlcCer, LacCer, and ceramide. In summary, we show that HIV and SIV envelope glycoproteins bind to GalCer, GlcCer, LacCer, and ceramide in a dose-dependent, saturable, and specific manner. These interactions may function as receptors of attachment in order to facilitate infection of CD4 low or negative cells or promote interactions with other receptors leading to the activation of signaling pathways or pathogenesis.

## 1. Introduction

The lipid composition of cell membranes is a determining factor in the sensitivity and permissivity of cells to certain viruses. Glycolipids seem to be strongly involved in the attachment of viruses to target cells and their penetration into these cells. Virus–glycolipid interactions have been revealed for several viruses by using direct molecular approaches to evaluate molecular virus–glycolipid binding [1,2] or in the presence of specific antibodies against selective glycolipids [1,2], or, indirectly, by blocking the biosynthetic pathways of certain glycolipids. For example, the depletion of sphingomyelin by treating cells with sphingomyelinase has underlined the importance of this sphingolipid in the attachment and entry, essentially of enveloped viruses belonging to different families, including the Ebola virus [3], influenza virus A [4], pseudorabies virus [5], Japanese encephalitis virus, and rubella virus [6]. More recently, the development of the CRISPR/Cas9 genome-wide knockout screening approach, by targeting glycolipid biosynthetic enzymes, has also revealed the strong involvement of glycolipids in host–virus interactions [6].

Due to their physicochemical structures, the glycolipids of membrane rafts have probably been selected by different pathogenic agents as platforms for attaching to and then penetrating target cells. These glycolipids, mainly localized with cholesterol at the level of the membrane rafts, play an essential role in cell physiology through their involvement in signaling pathways, cell growth, apoptosis, differentiation, and autophagy [7,8].

In the case of HIV-1, while the main receptor/coreceptors are CD4 and CCR5/CXCR4 [9,10], several attachment factors have been described, including Glycosaminoglycans (GAGs) and lectins [11]. CD4 and CCR5/CXCR4 are transmembrane glycoproteins, respectively related to the immunoglobulins and G-protein-coupled receptors. They are co-expressed on T-helper lymphocytes, monocytes, and macrophages in lipid raft domains, where they seem to establish some interactions with sphingolipids—at least with CD4 [12]. One of the important roles of these factors of attachment is to concentrate the virus at the cell surface in order to facilitate its encounter with the specific entry receptor, or to induce some structural modifications allowing it to recruit the entry receptor. Accordingly, it has been reported that several CD4-negative cells, such as cell lines originating in the nervous system [1,13], the intestine [14] and the liver [15], are susceptible to HIV-1 infection. These reports suggest an alternative entry pathway for HIV-1 that is CD4-independent. As a candidate, sphingolipid galactosylceramide (GalCer) has been reported to substitute for CD4 and promote HIV-1 infection in a CD4-independent mechanism [13,16]. GalCer is able to interact with HIV-1 gp120 and to act as a specific receptor for the infection of certain cell lines of neuronal origin, such as U373 MG and SK-N-MC [1,13,16]. Other authors have reported that the presence of GalCer on the intestinal epithelial cells of the jejunal tissue, which are GalCer+, CCR5+, CD4−, and CXCR4−, making them permissive to HIV-1 infection [17]. Thus, the high expression of GalCer and CCR5 by jejunal epithelial cells, which express neither CD4 nor CXCR4, would be the basis of the transmission of HIV-1 CCR5 tropic viruses during primo-infection via the mucosal route [17]. In this CD4-independent HIV-1 infection, GalCer or other sphingolipids could act as co-factors to induce structural modifications of HIV-1 glycoprotein, thus catalyzing the interaction with CCR5 and promoting viral entry. In agreement with this interpretation, it has been shown that GalCer expression alone on the intestinal epithelial HT29 cell line is not sufficient to promote HIV-1 entry, despite efficient virus attachment. Interestingly, these cells become permissive to HIV-1 following the co-expression of CXCR4 [18]. Glycolipids are used as receptors, not only by several other viruses, such as simian virus 40 (SV40) and murine polyoma virus (MPyV), both belonging to the polyomaviridae family [19,20], but also by bacteria and fungi [21]. Sphingolipids are involved in various biological functions including cell proliferation, differentiation, apoptosis, and signal transduction [7,8]. They are expressed ubiquitously in different tissues, with the exception of GalCer, which is more selectively present in the brain and kidneys and is also expressed on the plasma membrane of oligodendrocyte and Schwan cells [16]. In addition to binding viruses, glycosphingolipids are also used as receptors by various pathogenic bacteria, such as *Pseudomonas aeruginosa* [22], *Bordetella pertussis* [23], and helicobacter pylori [24], and also by fungal pathogens such as the opportunistic Candida albicans [23]. Adherence of *Pseudomonas aeruginosa* and Candida albicans to glycosphingolipid (Asialo-GM1) receptors is achieved by a conserved receptor-binding domain present on their adhesins [21]. Some bacterial toxins also use gangliosides and sphingolipids as receptors [25,26,27,28,29]. The ganglioside GM1 is used as a receptor by the cholera toxin (from *Vibrio cholerae*) [27], while the sphingolipid Gb3Cer (Globotriaosylceramide) serves as a receptor for Shiga toxin (from *Shigella dysenteriae*) [28]. These data highlight the importance of lipids in the tropism and mode of action of pathogens.

By using complementary biochemical and physicochemical approaches, the present study shows that, in addition to GalCer, the envelope glycoproteins of HIV-1 gp160, HIV-2 gp140, and SIV gp140 also interact with GlcCer, LacCer, and ceramide, ubiquitous glycolipids/lipids that could play an important role as receptors of attachment to promote the concentration of HIV-1, thus facilitating infection in a CD4-dependent or even CD4-independent manner, or promoting interaction with other receptors leading to activation of signaling pathways or pathogenesis.

## 2. Results

**Figure 1:**
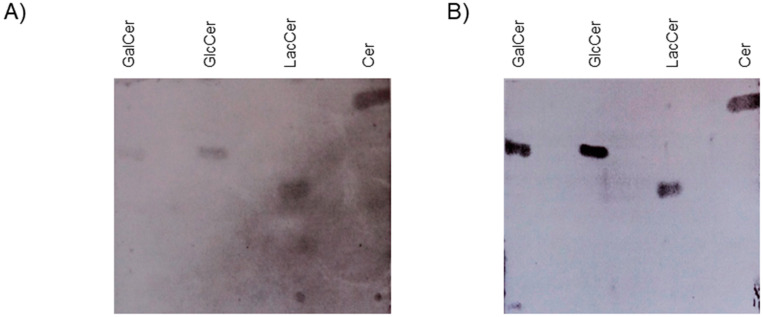
Binding of HIV-2 gp140 to glycolipids (GalCer, GlcCer, LacCer) and ceramide. (**A**) HPTLC-separated glycolipids/lipid were incubated with ^125^Igp140 from HIV-2. Lane 1: GalCer, Lane 2: GlcCer, Lane 3 LacCer, Lane 4: Ceramide. (**B**) The chromatographed lipids were visualized with iodine vapor. This figure is representative of 3 experiments.

**Figure 2:**
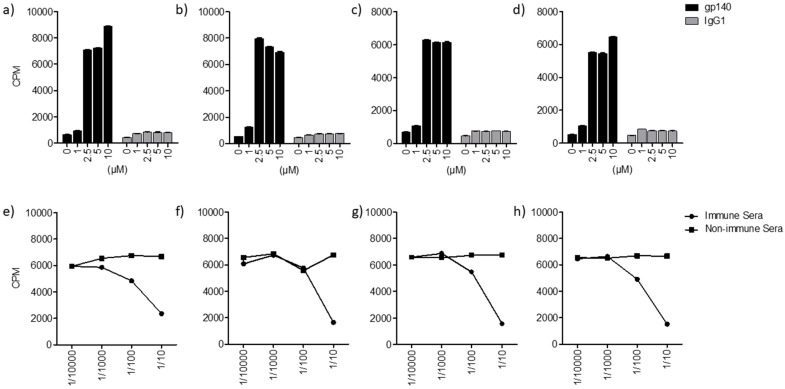
Interaction of lipids/glycolipids with HIV-2 ^125^I gp140. (**a**–**d**): Interaction of ^125^I gp140 from HIV-2 with GalCer (**a**), GlcCer (**b**), LacCer (**d**), and ceramide (**e**). (**e**–**h**): Inhibition of the gp140-glycolipds/lipid interactions by human anti-HIV-2 antiserum at the indicated dilutions. This figure is representative of 3 experiments. The error bars correspond to SD.

**Figure 3:**
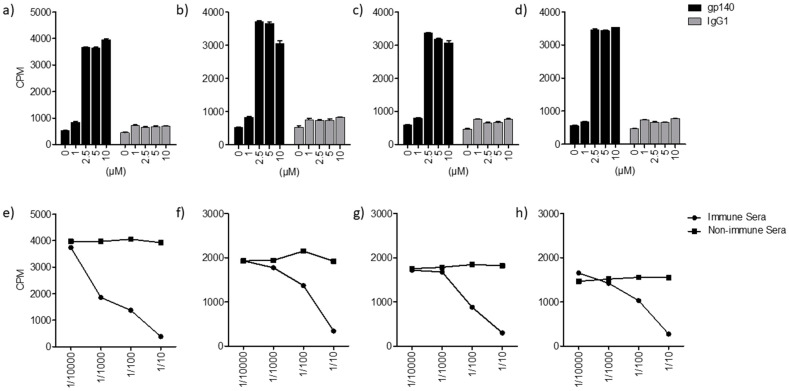
Interaction of glycolipids/lipid with SIV ^125^I gp140. (**a**–**d**): Interaction of ^125^I gp140 with GalCer (**a**), GlcCer (**b**), LacCer (**d**), and ceramide (**e**). (**e**–**h**): Inhibition of ^125^Igp140-glycolipds/lipid interactions by rhesus macaque anti-SIV antiserum or with pre-immune serum at the indicated dilutions. This figure is representative of 3 experiments. The error bars correspond to SD.

**Figure 4:**
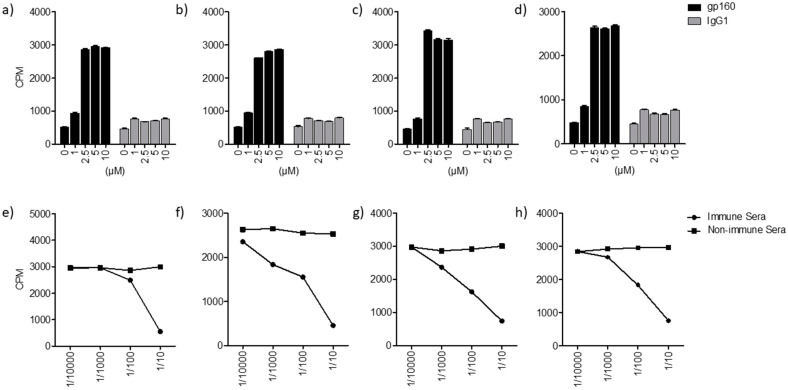
Interaction of lipids/glycolipids with HIV-1 125I gp160. (**a**–**d**): Interaction of 125I gp160 with GalCer (**a**), GlcCer (**b**), LacCer (**d**), and ceramide (**e**). (**e**–**h**): Inhibition of the gp160-glycolipds/lipid interactions by rabbit anti-gp160 antiserum or with pre-immune serum at the indicated dilutions. This figure is representative of 3 experiments. The error bars correspond to SD.

The interaction between HIV-2 glycoprotein (^125^I-gp140) and lipids/glycolipids was assessed using the HPTLC method as described in [1]. Briefly, after separation of glycolipids by thin layer chromatography (TLC), the gp–glycolipid interactions were analyzed by incubation of glycolipids (GalCer, GlcCer, LacCer) and ceramide, previously immobilized on silica gel plates, with ^125^I-gp140 from HIV-2. After autoradiography detection, a clear interaction between HIV-2 gp140 and the glycolipid GalCer, materialized by the revelation of a band that migrates with a mobility similar to that of HIV-2 gp140, was obtained (Figure 1A,B, lane 1). These results are in line with those previously reported for HIV-1 gp120 [1,16]. Interestingly, the experiments performed in this work also showed that HIV-2 ^125^I-gp140 interacted with the glycolipids GlcCer and LacCer (Figure 1A, lanes 2, 3). More interestingly, the results clearly show that ^125^I-gp140 also interacts with ceramide, indicating that the lipid moiety of GalCer, GlcCer, and LacCer is also implicated in the interaction with the HIV-2 envelope glycoprotein (Figure 1, lane 4). In addition, when the same experiments were conducted with ^125^Igp160 from HIV-1, similar results were obtained. To identify and determine the mobility of each glycolipid and lipid, the corresponding bands were visualized by revelation with iodine vapor (Figure 1A). Overall, using the same approach as previously reported in [1,16], our results demonstrate that the HIV-2 envelope glycoprotein is able to interact with GalCer, GlcCer, LacCer, and ceramide, and not only with GalCer as reported in [1,16]. It is interesting to note that the ceramide moiety of the ganglioside (globotriosyl ceramide) has been described for its role in the toxin–sphingolipid interaction [25]. Then, to further characterize these findings, the interactions between gp from HIV-1, HIV-2, and SIV and lipids/glycolipids were analyzed using two additional, complementary approaches. The first approach involved a solid-phase binding. Variable amounts of glycolipids/lipids, previously coated on a flexible 96-well microtitration plate, were incubated with a constant concentration of ^125^I-gp140 from HIV-2, ^125^I-gp140 from SIV, or ^125^I-gp160 from HIV-1. The results of these studies (Figure 2, Figure 3 and Figure 4) are in line with the HPTLC analysis and show that ^125^I-gp140 from HIV-2 interacts in a dose-dependent manner with GalCer (Figure 2a), GlcCer (Figure 2b), LacCer (Figure 2c), and ceramide (Figure 2d). The specificity of these interactions is demonstrated by the capacity of anti-gp140 antibodies (gp140 anti-sera) to inhibit these interactions in a dose-dependent manner. As a control, pre-immune sera (NI) used at the same dilutions did not interfere with these interactions (Figure 2e–h). Binding experiments with SIV ^125^I-gp140 (Figure 3a–h) and HIV-1 ^125^Igp160 (Figure 4a–h) with the same glycolipids/lipids led to the same conclusions, confirming a specific interaction of SIV and HIV-1 envelope glycoproteins with glycolipids GalCer, GlcCer, LacCer, and ceramide lipids (Figure 3a–h and Figure 4a–h). Antibodies against SIV gp140 and HIV-1 gp160, raised in SIV-infected macaques and gp160-immunized rabbits, totally inhibited these interactions (Figure 3e–h and Figure 4e–h), whereas pre-immune sera (NI) obtained from the same animal before infection or immunization had no effect on these interactions (Figure 3e–h and Figure 4E–H). It was shown that, when a mouse ^125^I IgG1 was used as a control instead of iodinated radiolabeled envelope glycoproteins, in the same conditions, no specific binding was observed with the glycolipids/lipids (Figure 2a–d, Figure 3a–d and Figure 4a–d). In conclusion, the analysis of the binding of HIV-1, HIV-2, and SIV glycoproteins by solid-phase radio-immunoassays clearly demonstrates, in line with the results of the analysis by HPTLC, the specific interactions of HIV and SIV envelope glycoproteins with GalCer, GlcCer, LacCer, and ceramide.

**Figure 5:**
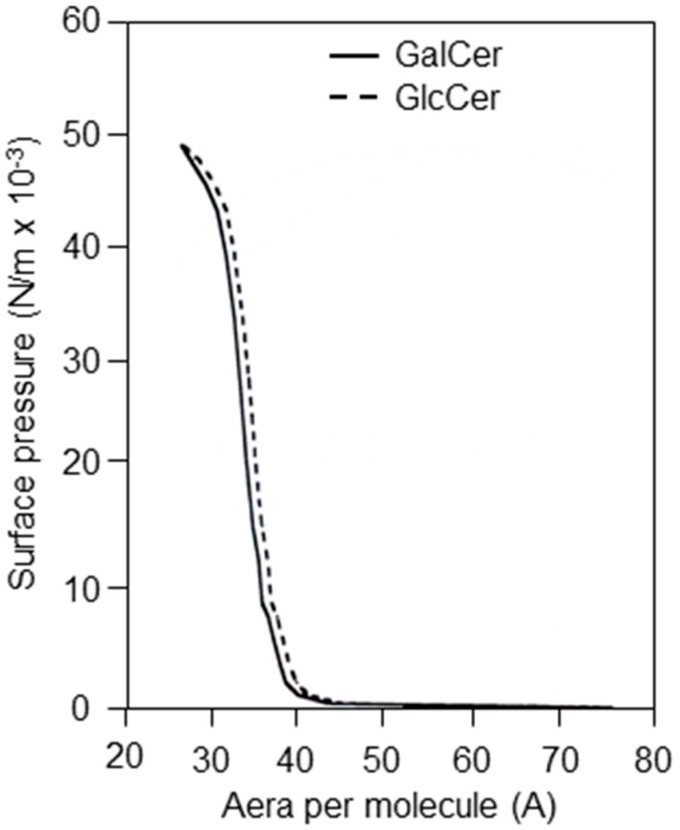
Surface pressure versus area (pi-A) isotherms of GalCer and GlcCer monolayers. Surface pressure was measured as a function of molecular area using a fully automated Langmuir balance. Glycolipids were dissolved at 1 mg/mL and were spread at the air/water interface with a glass capillary. This figure is representative of 3 experiments.

**Figure 6:**
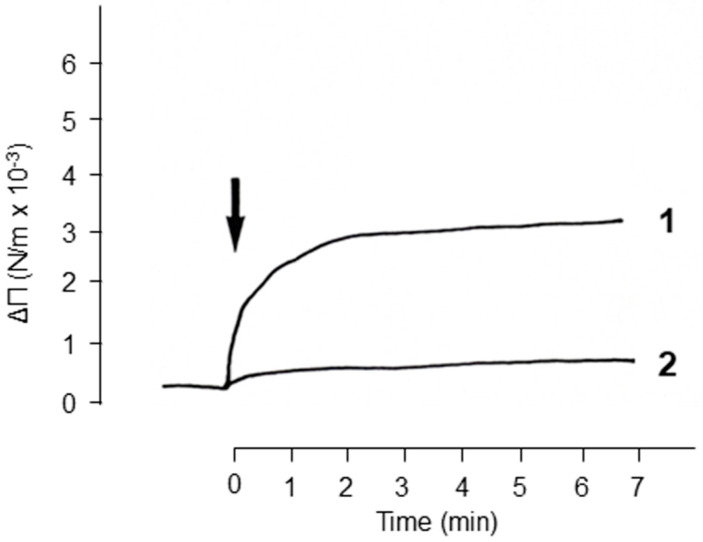
Interaction of HIV-1 gp160 glycoproteins with galactosylceramide structured as a monomolecular film. A monomolecular film of GalCer was established. The surface pressure was initially determined to be 6 mN/m by placing the platinum plate in the reaction compartment where the film was spread. The viral glycoprotein was then injected in the reaction compartment and kinetics were recorded. gp160 (330 ng) (Curve: 1) was injected in the aqueous phase (5 mL) composed of PBS buffer (pH 7.4). Increases in surface pressure was recorded with time. The control consisted of a solution of ovomucoid (2 µg) analyzed in the same conditions (Curve: 2). This figure is representative of 3 experiments.

**Figure 7:**
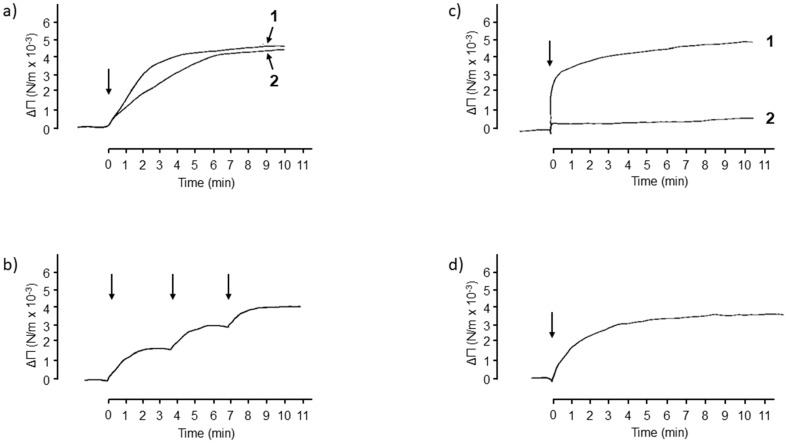
Interaction of HIV-1 gp160 with monomolecular films of glycolipids/lipid. (**a**) Interaction of HIV-1 glycoprotein gp160 with glycosyl ceramide. Comparative study of the interaction of HIV-1 gp160 with monomolecular films of GalCer (Curve: 1) and GlcCer (Curve: 2) initially stabilized at a surface pressure of 6 mN/m. (**b**) Kinetics study of the increase in surface pressure of GlcCer initially stabilized at a value of 6 mN/m after 3 successive additions of 330 ng of gp160. Each injection is shown by an arrow at time 0, 3.8, and 7 min. (**c**) Interaction of HIV1 gp160 glycoprotein with LacCer initially stabilized at a value of 6 mN/m. Kinetics of surface pressure increase after the addition of 330 ng of gp160 (Curve: 1); BSA (1.6 µg) was used as control (Curve: 2). (**d**) Interaction of HIV-1 gp160 glycoprotein with a monomolecular film of ceramide, initially stabilized at a value of 6 mN/m. Increases in surface pressures over time were recorded after adding 330 ng of gp160. This figure is representative of 3 experiments.

In the second approach, the interactions between envelope glycoproteins and glycolipids/lipids were evaluated in a more functional assay, based on the capacity of envelope glycoproteins to insert into a preformed, monomolecular layer film. The interactions of HIV-1 envelope glycoproteins (gp120/gp160) with different glycolipids structured as monomolecular layers were analyzed. Not all glycolipids and lipids can be structured in stable monomolecular films. So, the first step involved verifying that the glycolipids/lipids used here, GalCer, GlcCer, LacCer, and ceramide, were capable of structuring themselves in the form of a monomolecular film at the water/air interface. Figure 5 shows the surface pressure isotherms of the film (π) according to the molecular surface (Å) for the glycolipids GalCer and GlcCer. The increase in surface pressure was linear for these two glycolipids between 2.5 and 42 mN/m. The (π-Å) isotherms of these two glycolipids are classical, and characteristic curves of the formation of stable monomolecular films were obtained [30]. Initially, a GalCer monomolecular film was used to study the interactions of the glycolipids with HIV-1 gp120/gp160 glycoproteins. After stabilizing the monomolecular GalCer film at a surface pressure of 6 mN/m, glycoprotein gp160 was injected into the aqueous phase (PBS buffer pH 7.4). The addition of gp160 resulted in a rapid increase in surface pressure of 4 mN/m (Figure 6). This increase in the surface pressure of the GalCer film resulted from interactions of gp160 with the glycolipid GalCer. In a control of specificity, the addition of an unrelated glycoprotein, ovomucoid, caused no significant change in the surface pressure (Figure 6). The gp160–GalCer interactions shown by this method are in perfect agreement with the HPTLC binding results presented above and with those reported by Bhat et al. [16] and Harouse et al. [1]. However, they show, for the first time, to our knowledge, that HIV-1 envelope glycoproteins also interact with the monomolecular film of GlcCer, LacCer, and ceramide (Figure 7a–d). These interactions are specific, as shown by the incapacity of an unrelated protein bovine serum albumin (BSA) to interact with the glycolipids/lipids tested. Furthermore, the intensity of the interaction signal of gp160 with GlcCer varied in a dose-dependent manner with the gp160 concentrations. The successive additions of three doses of 330 ng of gp160 were accompanied by a rapid increase in the surface pressure (Figure 7b). These results are in correlation with those obtained in binding experiments between radiolabeled envelope glycoproteins and glycolipids or ceramide, and stress the importance of the lipid moiety in the interaction with HIV and SIV viral envelope glycoproteins.

## 3. Discussion

The primary pathway for HIV-1 binding and penetration is through CD4 and the CXCR4/CCR5 [9,10] coreceptors. However, several studies have shown alternative mechanisms based on CD4-independent pathways, involving alternative receptors such as glycospingolipids [1,16] and DC-SIGN [31]. In the present work, using different approaches, we showed that the binding of HIV and SIV glycoproteins to GalCer, GlcCer, LacCer, and ceramide is dose-dependent, saturable, and selectively blocked by specific anti-envelope glycoprotein antibodies. These findings suggest that, in addition to GalCer, other glycolipids/lipids, such as GlcCer, LacCer, and ceramide, are also able to interact specifically with HIV and SIV envelope glycoproteins. These interactions may function as receptors of attachment in order to facilitate the infection of target cells negative for CD4 and/or CCR5/CXCR4 coreceptors that are normally responsible for viral entry.

The results reported here, in agreement with the pioneering work of Harouse et al. [1] and Bhat et al. [16], show that HIV-1 glycoprotein gp160 interacts with GalCer. However, different conclusions can be drawn regarding the binding of HIV and SIV glycoproteins with other glycolipids as the present results show that the envelope glycoproteins of HIV-1, HIV-2, and SIV viruses are able to interact not only with GalCer but also with LacCer, GlcCer, and ceramide. These findings are supported by previous observations showing that gp120 of HIV-1 interacts significantly with LacCer and GlcCer [32,33]. An explanation for these different observations reported by the various groups could be related to the structural modification of the glycolipids and/or the viral glycoproteins encountered during the solid-phase assays. In accordance with these explanations, it has been shown that gp120 denaturation by heat treatment abolishes its capacity to interact with GalCer [33]. In contrast, its N-deglycosylation does not alter its capacity to interact with GalCer [33,34,35]. However, in our assays, the ability of HIV and SIV glycoproteins to interact with glycolipids does not appear to be impaired in the solid-phase, RIA, or TLC assays, or in the less denaturing liquid-phase monomolecular lipid assay. This interpretation is consistent with the ability of GalCer to interact with synthetic peptides mimicking the V3 loop, which has been identified as the gp120 domain involved in interaction with GalCer [36]. In addition to GalCer, V3 synthetic peptides are able to interact with other sphingolipids including LacCer, ceramide trihexoside, asialo-GM1, GM2, asialo-GM2, GM3, and GD3 [36]. It is important to note that, in our study, the concentrations of radiolabeled glycoproteins used in the assays (HIV-1 ^125^I-gp160 = 0.3 nM, HIV-2 ^125^I-gp140 = 0.7 nM, and SIV ^125^I-gp140 = 0.6 nM) are consistent with those reported in the sera of untreated HIV-1 infected patients [37].

Our results on monomolecular films of glycolipids show an interaction of envelope glycoprotein precursors (gp120 + gp41) of HIV-1, HIV-2, and SIV not only with GalCer as previously reported [38], but also with GlcCer, LacCer and ceramide. This difference could be related to the nature of the ligands used in this study, as the glycoproteins used contained both parts (SU_gp120_ + TM_gp41_), while those described in [38] were made only with surface glycoproteins (HIV-1 gp120, and HIV-2 gp105 SU). In agreement with this hypothesis, it has been reported that HIV-1 gp41, via its ELDKWA domain, would also be involved, in addition to the V3 region, in the interaction with GalCer [39,40]. This glycosphingolipid, GalCer, present on the surface of mucosal intestinal epithelial cells, seems to mediate the entry of HIV-1 by transcytosis at the level of CD4-negative epithelial cells [39,40].

In addition to GalCer, GlcCer, and LacCer, other GSLs (glycosphingolipids), including GM3 and GB3 gangliosides, have also been described for their capacity to interact with HIV-1 and also with various enveloped and non-enveloped viruses [38,41]. In agreement with this statement, treatment of CD4+ osteoblasts with the glucosylceramide synthase inhibitor 1-phenyl-2-hexadecanoylamino-3-morpholino-1-propanol (PPMP) has been shown to significantly alter the susceptibility of these cells to HIV-1 infection. This reduced susceptibility can be restored strongly or partially, by exogenous addition of GB3 or GM3, respectively, to the cell medium [42].

In the present study, we have shown that HIV and SIV envelope glycoproteins interact with GalCer, LacCer, GlcCer, and also with ceramide. Indeed, the involvement of ceramides in the viral cycles of several viruses has been reported to facilitate infection. Accordingly, increasing the level of intracellular ceramide by various approaches, including activation of serine palmitoyltransferase and ceramide synthase (two enzymes involved in the ceramide biosynthesis pathway [43]), enzymatic action with sphingomyelinase (Smase), or by exogenous addition of ceramide, leads to the inhibition of HIV-1 viral replication [44]. It is important to note that the inhibitory effect obtained in the presence of Smase suggests the involvement of cell surface ceramide in HIV-1 infection [44]. Other reports have also highlighted, directly or indirectly, the importance of ceramide concentrations in controlling infection of several other viruses including Ebola virus [3], hepatitis C virus [45], the Japanese encephalitis virus [46], HIV-1 [47,48], and SARS-CoV2 [49]. It is interesting to note that certain antidepressants, including amitriptyline, sertraline, and fluoxetine, known for their inhibitory action on the ceramide generation pathway by blocking the acid sphingomyelinase, block the replication of SARS-CoV-2 [50].

In summary, we have shown, using different approaches, that the binding of HIV and SIV glycoproteins to GalCer, GlcCer, LacCer, and ceramide is dose-dependent, saturable, and selectively blocked by specific anti-envelope glycoprotein antibodies. These findings suggest that, in addition to galactosylceramide, other glycolipids/lipids such as GlcCer, LacCer, and ceramide are also able to interact specifically with HIV and SIV envelope glycoproteins. These interactions may function as receptors of attachment in order to facilitate infection of reduced CD4 cells or CD4-negative cells expressing the coreceptors CCR5/CXCR4, which are responsible for the viral entry mechanism, or promote interaction with other receptors, leading to signaling pathway activation or pathogenesis.

As therapeutic applications, these lipids could be considered as targets in the treatment of viral infections. This could be envisaged essentially by targeting the enzymes involved in the metabolic pathways concerning the biosynthesis of these lipids.

## 4. Materials and Methods

Envelope glycoproteins and anti-envelope antibodies: HIV-1 glycoproteins gp120 and gp160 were obtained as recombinant proteins in the vaccinia virus/BHK-21 cell system as described elsewhere by Fenouillet et al. [51]. SIV recombinant gp140 of SIVmac251 was obtained as a gift from Pasteur Mérieux Sérum et Vaccin (Val de Reuil, France). The env coding sequence was deleted from the transmembrane domain (Ile693—Leu716), mutated at the furan cleavage site, and introduced as transgene in the vaccinia virus. The soluble gp140 was produced in transduced BHK cells and purified as described [52]. HIV-2 gpl40 [53] was produced by a similar protocol and purified from the cell supernatant by adsorption on heparin-Ultrogel followed by gel filtration on Sephacryl S 300 and anion exchange HPLC as described in [54].

Anti-gp160 antibodies were produced in rabbit as described in [54]. Pre-immune sera (NI), collected before immunization, was used as control, and immune sera (I) collected after the last immunization was used as a source of anti-envelope antibodies.

Glycolipids were purchased from Sigma-Aldrich. GalCer (C2137), LacCer (427572), GlcCer (C8752), and ceramide (C1516) were purified from bovine brain with a homogeneity >98%.

Radiolabeling of envelope glycoproteins: Radiolabeling of envelope glycoproteins, HIV-1 gp160, HIV-2 gp140, and SIV gp140, with ^125^I was performed as described in [51], and obtained with a radioactive specificity between 35 and 40 µCi/µg.

Binding of HIV-2 gp140 to glycolipids (GalCer, GlcCer, LacCer) and ceramide: Glycolipids were separated on a HPTLC plate (alumina-backed silica gel G60, Merck, Darmstadt, Germany) in a mixture of CHCl_3_/CH_3_OH/H_2_O (65/25/4). The HTPLC plates were saturated with 5% milk in PBS buffer for 1 h at room temperature, then washed once with PBS buffer. Recombinant gp140 was radiolabeled with [^125^I] as previously described [55]. The TLC plate was incubated with 6.10^6^ cpm/mL of ^125^I-gp140 in 15 mL of PBS overnight at room temperature. The plate was washed 3 times for 10 min with PBS, then exposed to X-OMAT film (Kodak, Sigma-Aldrich, St Louis, MO, USA) for 26 h. HTPLC chromatographed lipids were identified by iodine vapor treatment.

Solid-phase binding assay: Lipids dissolved in a mixture of CHCl_3_/CH_3_OH (2/1) were adsorbed on a microtitration plate (Microtest III Flexible, Becton Dickenson, Oxnard, CA, USA)) until the solvent evaporated. The plates were saturated with 5% milk for 1 h at 37 °C and then washed 3 times with 0.1% Tween 20 in PBS. ^125^I-gp140 (0.2 × 10^6^ cpm/well) was added. Plates were then incubated for 2 h at 37 °C and washed 7 times with 0.1% Tween 20 in PBS. Wells were cut and radioactivity was determined with a gamma counter (Tricarb Packard, Diamond Hill Road, Woonsocket, USA).

Inhibition of ^125^I-gp140-glycolipds/lipid interactions were performed with human anti-HIV-2 antiserum. ^125^I-gp140 was previously incubated with various dilutions of antiserum or with non-immune serum at the indicated dilutions for 2 h at 37 °C. Inhibition of the SIV ^125^I-gp140-glycolipds/lipid interactions were performed with rhesus macaque anti-SIV antiserum or with pre-immune serum at the indicated dilutions for 2 h at 37 °C. Inhibition of the HIV-1 ^125^I-gp160-glycolipds/lipid interactions was challenged with rabbit antigp160 antiserum or with pre-immune serum at the indicated dilutions for two hours at 37 °C.

Functional assays—surface pressure versus area (pi-A) isotherms of GalCer and GlcCer monolayers: Surface pressure was measured as a function of molecular area using a fully automated Langmuir balance as described by Moreau et al., [56]. Glycolipids were dissolved at 1 mg/mL and were spread at the air/water interface with a glass capillary. GalCer, GlcCer, LacCer, and ceramide were purchased from Sigma-Aldrich. The lipids were homogeneous as shown by thin layer chromatography and visualized with orcinol.

Glycoprotein interactions with glycolipids/lipid in monomolecular film—Interaction of HIV-1 gp160 glycoprotein with galactosylceramide structured as a monomolecular film: A monomolecular film of GalCer was established. The surface pressure was initially determined to be 6 mN/m by placing the platinum plate in the reaction compartment where the film was spread. The viral glycoprotein was then injected in the reaction compartment and kinetics were recorded as described by Verger and de Haas [57]. Glycoprotein gp160 was injected in the aqueous phase (5 mL) composed of PBS buffer (pH 7.4). Increases in surface pressure was recorded over time. The control consisted of a solution of ovomucoid (2 µg) analyzed in the same conditions.

## Data Availability

All data generated or analyzed during this study are included in this published article.
